# T191. RANDOMIZED DOUBLE-BLIND FEASIBILITY STUDY OF A GLUTEN-FREE DIET IN PEOPLE WITH SCHIZOPHRENIA AND ELEVATED ANTIGLIADIN ANTIBODIES (AGA IGG)

**DOI:** 10.1093/schbul/sby016.467

**Published:** 2018-04-01

**Authors:** Deanna Kelly, Haley Demyanovich, Katrina Rodriguez, Daniela Cihakova, Monica Talor, James Gold, Sharon August, Robert Buchanan, Stephanie Feldman, Fang Liu, William Carpenter, William Eaton

**Affiliations:** 1 University of Maryland Baltimore, Maryland Psychiatric Research Center; 2 Johns Hopkins Bloomberg School of Public Health

## Abstract

**Background:**

Deconstructing schizophrenia is important, since different disorders within the syndrome may have distinct pathophysiological mechanisms and treatment targets. Emerging evidence suggests inflammation may play a role in at least a subgroup of people with this illness. One specific subgroup with known inflammation is a group with elevations in antigliadin antibodies (AGA IgG). These elevations (>20 U) are found in about 1/3 of people with schizophrenia. Gliadin is a protein found in wheat, barley and rye. This subgroup with AGA IgG elevations may be distinct as they have fewer positive symptoms, higher kynurenic acid levels, and may benefit from a gluten free diet. The effectiveness of gluten removal has been controversial with mixed results in previous studies, however no former study has examined gluten removal in those with high AGA IgG, that is, the population who may be expected to benefit.

**Methods:**

Sixteen people with a DSM-IV-TR diagnosis of schizophrenia or schizoaffective disorder and who also had elevated AGA IgG (≥20 U) were recruited and admitted to an inpatient unit for a 5-week randomized double blind trial. Participants were randomized in a double blind fashion to either a diet containing gluten or a complete strictly enforced gluten free diet. This was accomplished by given 10 gm of gluten flour or 10 gm of rice flour daily in a protein shake, with all meals for all participants being standardized and gluten free. Participants had cooking classes, received cookbooks, and went on shopping trips for gluten free foods and meals. Participants were rated on the Brief Psychiatric Rating Scale (BPRS), Scale for the Assessment of Negative Symptoms (SANS) and the MATRICS Cognitive Battery (MCCB) at baseline and 5 weeks. The study was not powered to find a treatment effect, but designed to examine the feasibility of conducting an inpatient gluten removal study and examine trends in treatment as measured by Cohen’s D effect size (ES) differences.

**Results:**

Fourteen of 16 people completed the 5-week trial and all tolerated the diet (2 discontinued early in the trial for housing and administrative reasons). The mean age of participants was 37.9 ± 13.2 years, 56% male and 75% African-American. During the clinical trial, participants receiving the gluten free diet had an improvement in negative symptoms as compared to placebo (treatment difference) with an ES=0.53. There was no improvement in BPRS total score or positive symptoms. The MCCB composite score did not improve, but an ES=0.6 was noted in the domain of attention favoring the gluten free diet. The AGA IgG levels decreased by 35% in the five weeks in the gluten free diet group relative to a 17% decrease in the gluten containing group. It is also important to note that the correlation between the change in SANS total score and AGA IgG in the gluten free group (r=0.57) was strong and notably different than the correlation between the change in SANS total score and AGA IgG in the gluten containing group (r=-0.017), suggesting a possible marker of treatment effect. Adverse effects were similar between treatment groups.

**Discussion:**

This is the very first study of a gluten free diet in schizophrenia with elevated AGA IgG. This feasibility study suggests that removal of gluten is associated with improvement in negative symptoms and attention, but not positive symptoms. Participants tolerated the diet. The feasibility study provided data to design the now ongoing fully powered confirmatory double-blind trial in people with schizophrenia with negative symptoms using a higher gluten amount (30 grams daily) and with aims to examine associated mechanisms, with targets of inflammation, neuroimaging and gut permeability.

